# Sedentary-related abdominal fat accumulation reduced by administrating heat-treated *Bacillus subtilis*-derived postbiotic

**DOI:** 10.3389/fnut.2025.1612804

**Published:** 2025-07-15

**Authors:** Mi Wang, Feng He, Weishuang Meng, Zeliang Chen, Huijie Yang, Shi Qi Xu, Dang De Xin, Desheng Li

**Affiliations:** ^1^College of Animal Husbandry and Veterinary Medicine, Jinzhou Medical University, Jinzhou, China; ^2^Liaoning Kaiwei Biotechnology Co., Ltd., Jinzhou, China; ^3^Collaborative Innovation Center for Prevention and Control of Zoonoses, Jinzhou Medical University, Jinzhou, China; ^4^Liaoning Inspection Examination & Certification Center, Shenyang, China; ^5^Institute of Physical Education, Hubei University of Arts and Science, Xiangyang, China; ^6^Department of Endocrinology, Xiangyang Central Hospital, Affiliated Hospital of Hubei University of Arts and Science, Hubei University of Arts and Science, Xiangyang, China

**Keywords:** phosphatidylcholine, 13(S)-HODE, lipid metabolism, postbiotic, intestinal microbiota, metabolomics

## Abstract

**Background:**

A sedentary lifestyle can induce obesity, characterized by the accumulation of abdominal fat. Due to contemporary breeding practices, developing chicks exhibit increased resting time and decreased locomotor activity, resembling the sedentary lifestyle of humans. Developing chicks often show a substantial accumulation of abdominal fat and possess a digestive tract structure similar to humans. Consequently, they are widely used as experimental animal to study measures for improving intestinal health and reducing abdominal fat accumulation.

**Methods:**

In this study, we used chicks as experimental animal to investigate the effects of administering heat-treated *B. subtilis*-derived postbiotic (high-temperature treated *B. subtilis*) on abdominal fat accumulation, hematology parameters, intestinal microbiota composition, and intestinal contents and serum metabolites composition. A total of 120 day-old chicks were randomly assigned to two groups (CON; IBS) based on their initial body weight (52.79 ± 0.60 g). Each group had 6 replicates, with each replicate containing 10 animals. Animals in the IBS group were administered 0.30% heat temperature treated-*B. subtilis* for 42 days. On the final day, three animals were selected from each replicate to collect abdominal fat and liver organs, serum samples, and cecal content samples for further analysis.

**Results:**

The results indicated that administering the postbiotic reduced abdominal fat accumulation, as well as serum total cholesterol and triglyceride levels. Moreover, heat-treated *B. subtilis*-derived postbiotic administration decreased the abundance of *Bacteroides*, *Sphingomonas*, and *Klebsiella*, while increasing *Streptococcus*, *Veillonella*, *Allobaculum*, and *Dorea* in the intestine. Metabolomic analyses revealed that administering the postbiotic reduced intestinal phosphatidylcholine and serum 13(S)-HODE levels. Spearman correlation analysis suggested a potential link between *Klebsiella* and *Sphingomonas* bacteria and these metabolites.

**Discussion:**

As phosphatidylcholine plays a key role in facilitating intestine to absorb lipids from diet, administering heat-treated *B. subtilis*-derived postbiotic was therefore to be considered as an effective measure in regulating intestinal microbiota composition and their lipid metabolic activity, thereby controlling the development of obesity.

## 1 Introduction

Sedentary behavior can lead to obesity, which is a risk factor for many chronic diseases (1). Recent advances in the understanding of the gut-adipose tissue axis have highlighted the significant role of gut microbiota in lipid metabolism (2). Several studies have demonstrated a strong correlation between gut microbes and obesity (3, 4). Duan et al. (5) noted that abnormal lipid metabolism in obese subjects was associated with a decrease in the diversity of gut microbiota. A study by Xu et al. (6) demonstrated that applying exogenous measures to ameliorate the gut microbiota imbalance induced by feeding with a high fat diet was beneficial in decreasing fat accumulation and improving abnormal lipid profiles. Hence, strategies focusing on modulating gut microbiota composition could be key to controlling obesity development.

Dietary intervention through *B. subtilis* administration has emerged as an effective method to modulate intestinal microbiota composition (7, 8) and control obesity development (9, 10). It has been realized that not only viable microorganisms, but inactivated probiotics, known as ‘postbiotics’, can also confer health benefits (11, 12). However, heat-treated *B. subtilis*-derived postbiotic, as a postbiotic derived from *B. subtilis*, still has limited understanding of its ability to regulate lipid metabolism. Studies investigating other probiotic-derived postbiotics have provided promising insights. Yoshitake et al. (13) reported that administering heat-killed *L. plantarum* L-137 would ameliorate abnormal blood lipid metabolism induced by feeding with a high fat diet. Kikuchi et al. (14) noted that feeding a high fat diet and providing *B. longum* BR-108-derived postbiotic can reduce epididymal body fat mass and ameliorate abnormal blood lipid metabolism. Watanabe et al. (15) demonstrated that administering heat-killed *L. brevis* KB290 would decrease high fat diet feeding-related epididymal and renal adipose tissue weight increase through regulating intestinal microbiota composition. Additionally, heat-killed *L. plantarum* K8 also presents similar positive effects on ameliorating high fat diet feeding-related white adipose tissue hypertrophy and hepatic fat accumulation and abnormal blood lipid metabolism (16, 17). Compared with other probiotics, *B. subtilis* has the characteristics of simple culture conditions and fast proliferation (18). Therefore, the preparation cost of heat-treated *B. subtilis*-derived postbiotic is lower. In addition, the metabolites derived from probiotics mainly depend on the metabolites of probiotics, while *B. subtilis* has rich coding genes and can produce a variety of bioactive substances (organic acids, polysaccharides, etc.) (19, 20). Therefore, administering heat-treated *B. subtilis*-derived postbiotic may also have the potential to generate positive effects on regulating lipid metabolism and controlling obesity development.

We hypothesized that administering heat-treated *B. subtilis*-derived postbiotic would reduce sedentary-related abdominal fat accumulation by regulating the composition of intestinal microbiota and their lipid metabolic activity. Chicks served as the experimental animals in this study because contemporary poultry breeding practices have led them to tend to rest and show decreased locomotor activity (21). Moreover, avian abdominal fat accumulation patterns and digestive tract structures share similarities with mammals (22, 23). The objective of this study was to investigate the effects of administering heat-treated *B. subtilis*-derived postbiotic on abdominal fat accumulation, hematology parameters, intestinal microbiota composition, and intestinal contents and serum metabolites composition.

## 2 Materials and methods

### 2.1 Ethics statement

The experimental protocol was approved and overseen by the Animal Care and Use Committee of Jinzhou Medical University (Jinzhou, China) (protocol number JAU20250120).

### 2.2 Preparation of heat-treated *B. subtilis*-derived postbiotic

The strain of *B. subtilis* ACCC 11025 was incubated in lysogeny broth at 37°C for 24 h. After incubation, the culture was subjected to centrifugation at 11,000 × *g* for 10 min, followed by two washes. The obtained suspension underwent autoclaving at 121°C for 15 min, and the inactivation of bacterial cells was confirmed by the lack of bacterial growth on nutrient agar plate at 37°C for up to 72 h. The final product was processed using the spray-drying method.

### 2.3 Experimental design

A total of 120 day-old chicks were randomly assigned to two groups (CON; IBS) based on their initial body weight (52.79 ± 0.60 g). Each group had 6 replicates, with each replicate containing 10 animals. Animals in the IBS group were administered 0.30%heat-treated *B. subtilis*-derived postbioti for 42 days. All animals were managed in the same way, except for dietary treatments (24). Throughout the experimental period, the animals were provided unrestricted access to both feed and water. The nutritional requirements of the feed formula were optimized from the recommendations by the National Research Council and successfully applied in commercial (Boin Feed Company, Shenyang, China) (Supplementary Table 1).

On the final day, three animals were selected from each replicate based on the average body weight (2987.55 ± 86.04 g) to collect serum samples from the wing vein. Subsequently, these animals were euthanized by intravenously administering 1 cc of Euthasol to obtain abdominal fat and liver organs and cecal content samples for further analysis.

### 2.4 Parameters measurement

#### 2.4.1 Hematology parameters

On the final day, selected animals were used to collect blood samples from the wing vein. The collected blood samples underwent centrifugation at 3,000 × *g* at 4°C for 15 min to isolate the serum, which was then stored in duplicate. Subsequently, serum concentrations of total cholesterol, triglycerides, total bilirubin, gamma-glutamyl transferase, alanine aminotransferase, and total bile acids were analyzed using a fully automated biochemical analyzer (SMT-120VP, Seamaty, Chengdu, China).

#### 2.4.2 Organ indexes

After blood collection, animals were euthanized by intravenously administering 1 cc of Euthasol. The abdominal fat and liver were then removed and weighed to calculate the relative organ weight using the formula below:

$$\text{Organ index}=\frac{\text{Organ weight}}{\text{Live body weight}}\times 100\%.$$

#### 2.4.3 Intestinal microbiota analysis

Cecal contents from the sampled animals underwent DNA extraction using a magnetic Soil and Stool DNA kit (cat# DP712, TIANGEN Biotech Co., Ltd., Beijing, China). The concentration and purity of the extracted DNA were assessed using a Qubit 2.0 spectrophotometer (Invitrogen, Carlsbad, CA) and 1% (w/v) agarose gel electrophoresis. Following extraction, DNA samples were diluted to a concentration of 1 ng/μL with sterile water and stored at −20°C until analysis. For microbial community analysis, the V3-V4 hypervariable regions of the bacterial 16S rRNA gene were amplified using specific full-length universal forward (5’-ACTCCTACGGGAGGCAGCA-3′) and reverse (5’-GGACTACHVGGGTWTCTAAT-3′) primers. PCR reactions were performed in triplicate, and resulting products were purified using a Qiagen Gel Extraction Kit (cat# 28706, Qiagen, Germany). The purity of the PCR mixture was confirmed using a Qubit 2.0 dsDNA HS Assay Kit (cat# Q32854, Invitrogen). Microbial community structures were analyzed through 16S rRNA sequencing on the NovaSeq 6,000 platform (Illumina, San Diego, CA) at Shanghai Personal Biotechnology Co., Ltd. (Shanghai, China).

To ensure accuracy and reliability, the raw sequencing data underwent several processing steps. Firstly, Cutadapt software version 1.9.1 was employed to eliminate low-quality reads, and chimeric sequences were trimmed through alignment and detection processes. Subsequently, the remaining high-quality reads were clustered into operational taxonomic units (OTUs) at a sequence identity of 97% using Uparse v7.0.1001.

Taxonomic assignment of representative sequences was conducted using QIIME v1.9.1. To evaluate microbial diversity, rarefaction curves were generated for each sample in R software (version 1.9.1) to determine the suitable sequencing depth that captures the full extent of microbial diversity. Various alpha-diversity metrics, including Chao1, Pielou_e, Shannon, and Simpson diversity indices, were calculated based on the number of observed OTUs. Furthermore, beta-diversity analysis was carried out using the Jaccard distance metric.

#### 2.4.4 Metabolomics analysis

Homogenized samples were combined with methanol/acetonitrile (1:1, v/v) and subjected to centrifugation for 15 min at 14,000 × g and 4°C. Following centrifugation, the resulting supernatant underwent drying in a vacuum centrifuge. Subsequently, the dried samples were reconstituted in 100 μL acetonitrile/water (1:1, v/v) for metabolite detection using an UHPLC system (1,290 Infinity LC, Agilent Technologies) coupled to a quadrupole time-of-flight (AB Sciex TripleTOF 6,600). The HILIC separation was performed on an ACQUITY UPLC BEH 1.7 μm column (Waters, ACQUITY UPLC BEH Amide), with an ESI source utilized in both positive (POS) and negative (NEG) ionization modes. The mobile phase, consisting of 25 mM ammonium acetate and 25 mM ammonium hydroxide in water (A) or acetonitrile (B), underwent a gradient starting at 85% B for 1 min, linear reduction to 65% over 11 min, further reduction to 40% for 0.1 min, maintenance for 4 min, and a final increase to 85% over 0.1 min, with a 5-min re-equilibration. For RPLC separation, an ACQUITY UPLC HSS T3 1.8 μm column was employed, with water containing 0.1% formic acid (A) and acetonitrile with 0.1% formic acid (B) used as the mobile phase in positive mode, and 0.5 mM ammonium fluoride added to both water (A) and acetonitrile (B) in the mobile phase for negative mode. The flow rate was maintained at 0.3 mL/min, and the column temperature was set at 25°C. In MS/MS phase, the instrument covered the m/z range of 25–1,000 Da, with an accumulation time of 0.05 s/spectrum for the TOF MS scan.

To obtain mass-to-charge ratio, retention time, and peak area based on positive and negative ion models, we conducted the identification, filtering, and alignment of peaks. The raw data underwent transformation into mzXML format using ProteoWizard, and the XCMS project was utilized for peak alignment, retention time correction, and peak area extraction. The identification of peaks corresponding to metabolites was achieved through matching with the mzCloud database^1^. For the creation of a mass spectral library, Thermo mzVault (version 2.3) was employed. Quantification of the identified metabolites was performed using Tracefinder (version 4.1).

### 2.5 Statistical analysis

All collected data were subjected to normality testing using the Shapiro–Wilk test and quantile-quantile plots to ensure adherence to normal distribution. Each replicate was considered an experimental unit for analysis. Student’s t-test conducted using SAS software (version 9.4) was applied to assess various parameters. A probability value below 0.05 was considered statistically significant.

For metabolomics analysis, we utilized Orthogonal Projection to Latent Structures-Discriminant Analysis (OPLS-DA) to uncover variations in metabolites among groups. The screening of potential metabolites relied on both the Variable Importance in the Projection (VIP) value obtained from OPLS-DA and the *p*-value from a Student’s t-test. Significance was attributed to metabolites with a VIP value exceeding 1 and a *p*-value less than 0.05, ensuring a rigorous identification of statistically significant metabolites.

## 3 Results

**Figure 1:**
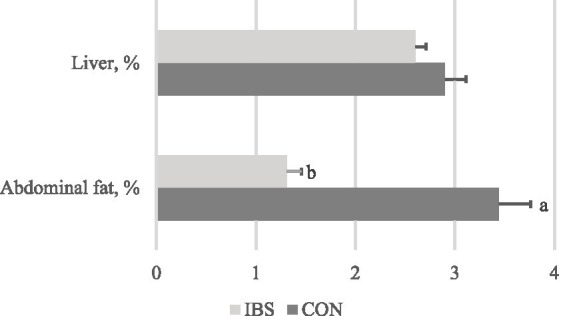
Effects of administrating heat-treated *B. subtilis*-derived postbiotic on organ indexes. Values represent the means of 6 replicates per group (*n* = 6). CON group was not administrated with any exogenous factors IBS group was administrated with 0.30% heat-treated *B. subtilis*-derived postbiotic. ^a,b^ Different superscripts between columns indicate significant difference (*p* < 0.05).

We found that administering heat-treated *B. subtilis*-derived probiotics reduced the relative weight of abdominal fat by 62.2% times (*p* < 0.001), but did not affect the liver (Figure 1).

**Figure 2:**
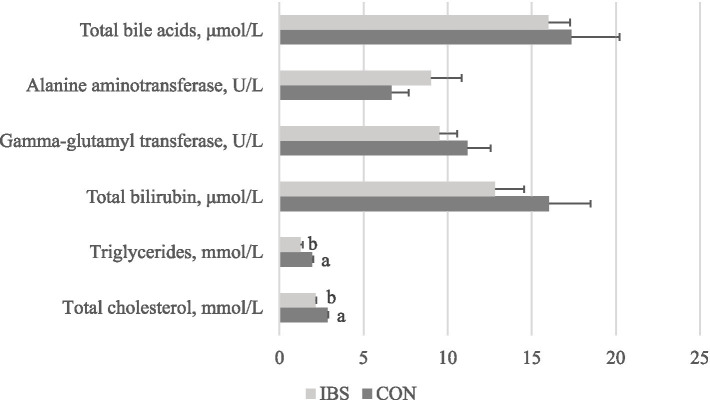
Effects of administrating heat-treated *B. subtilis*-derived postbiotic on hematology parameters. Values represent the means of 6 replicates per group (*n* = 6). CON group was not administrated with any exogenous factors IBS group was administrated with 0.30% heat-treated *B. subtilis*-derived postbiotic. ^a,b^Different superscripts between columns indicate significant difference (*p* < 0.05).

We found that giving heat-treated *Bacillus subtilis*-derived probiotics reduced triglycerides by 25% (*p* < 0.001) and total cholesterol by 35.5% (*p* = 0.003). However, the levels of total bilirubin, glutamyl transferase, alanine transferase, and total bile acids did not differ among groups (Figure 2).

**Figure 3:**
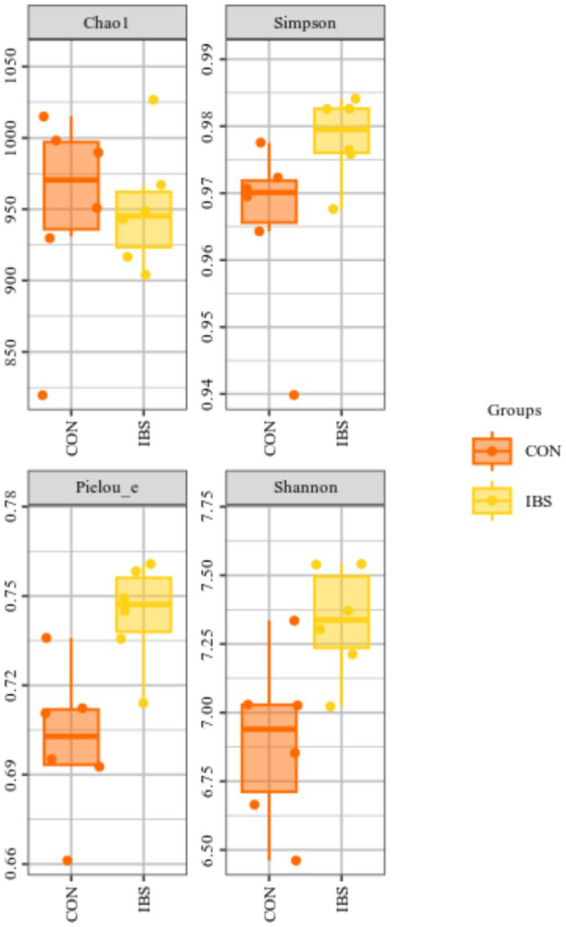
Effects of administrating heat-treated *B. subtilis*-derived postbiotic on alpha-diversity of intestinal microbiota, identified by 16S rRNA sequencing technique. CON group was not administrated with any exogenous factors IBS group was administrated with 0.30% heat-treated *B. subtilis*-derived postbiotic.

The alpha-diversity of the intestinal microbiota revealed that administering heat-treated *B. subtilis*-derived postbiotic significantly increased Pielou_e index (*p* = 0.007) and Shannon index (*p* = 0.015) (Figure 3)”.

**Figure 4:**
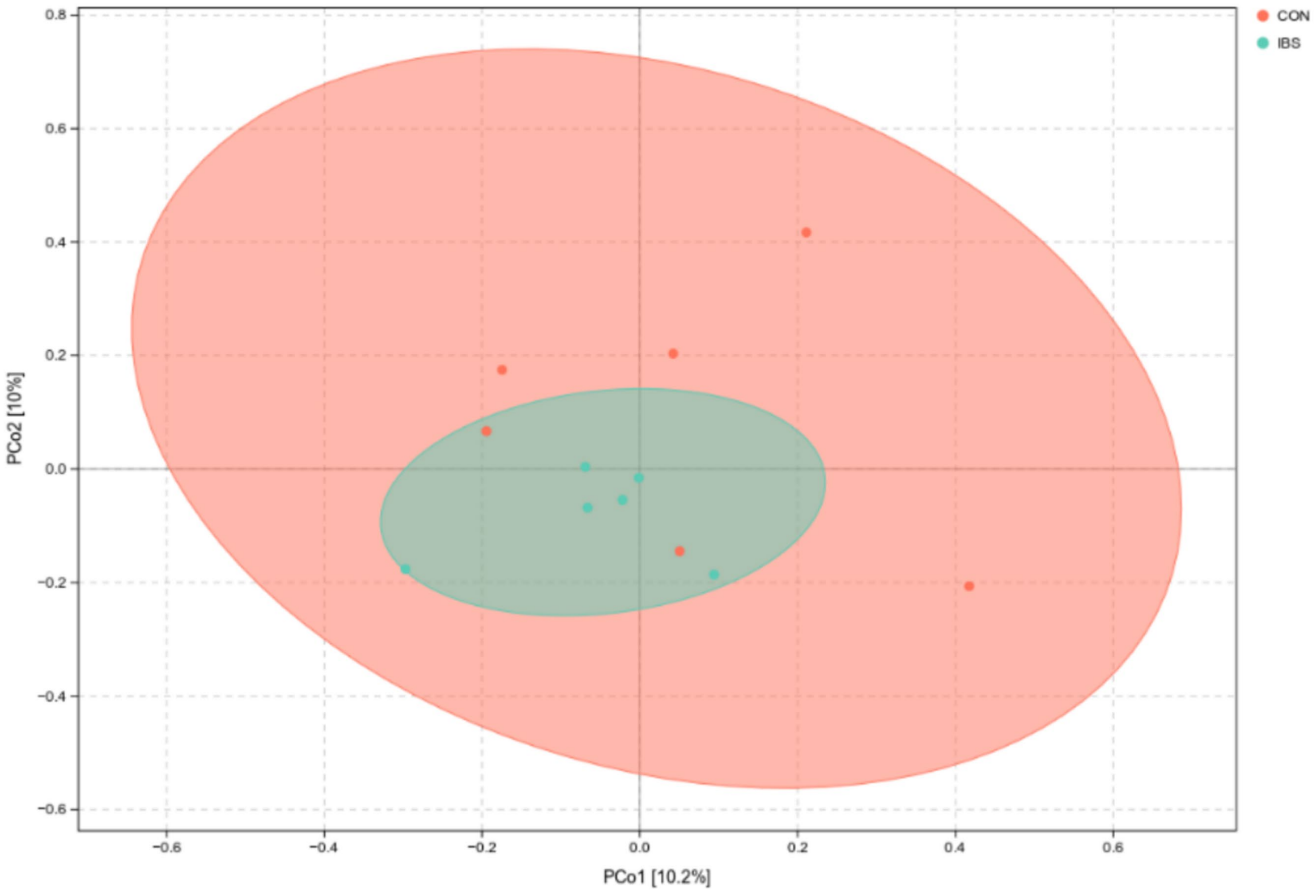
PCoA plot for intestinal microbiota as affected by administrating heat-treated *B. subtilis*-derived postbiotic, identified by 16S rRNA sequencing technique with jaccard distance algorithm. CON group was not administrated with any exogenous factors IBS group was administrated with 0.30% heat-treated *B. subtilis*-derived postbiotic.

However, the beta-diversity of the intestinal microbiota, as displayed in the PCoA diagram, did not show two completely separated circles. This indicates that beta-diversity among the intestinal microbiota did not differ among groups (Figure 4).

**Figure 5:**
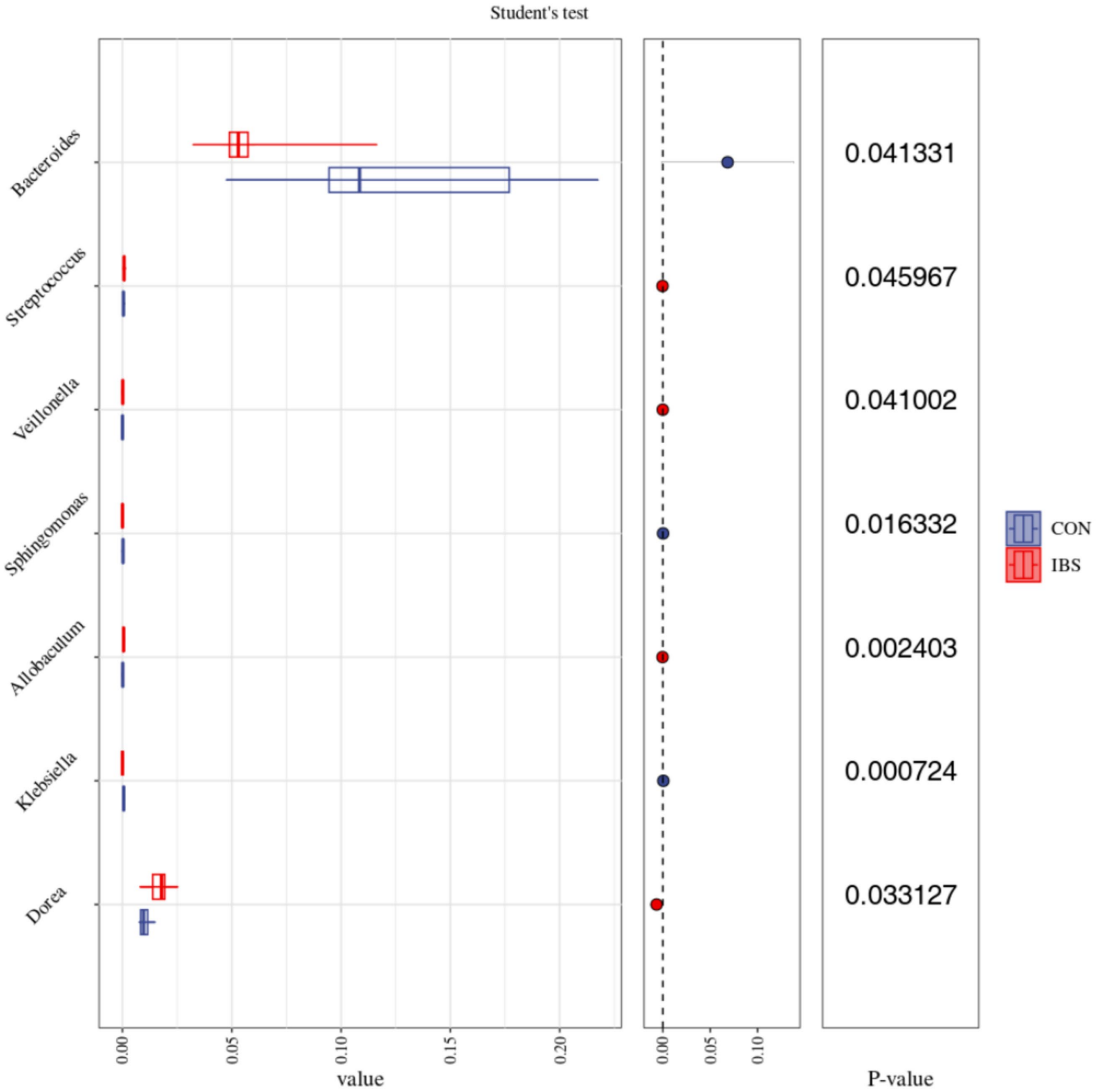
Effects of administrating heat-treated *B. subtilis*-derived postbiotic on intestinal microbiota on Genus level, identified by 16S rRNA sequencing technique and analyzed by Student’s t test statistical method. Values represent the means of 6 replicates per group (*n* = 6). CON group was not administrated with any exogenous factors IBS group was administrated with 0.30% heat-treated *B. subtilis*-derived postbiotic.

We further analyzed the differences in intestinal microbiota among groups at the genus level (Figure 5). Our findings revealed that the administration of heat-treated *B. subtilis*-derived postbiotic significantly reduced the abundance of *Bacteroides* (*p* = 0.041), *Sphingomonas* (*p* = 0.016), and *Klebsiella* (*p* < 0.001), while increasing the abundance of *Streptococcus* (*p* = 0.046), *Veillonella* (*p* = 0.041), *Allobaculum* (*p* = 0.002), and *Dorea* (*p* = 0.033).

**Figure 6:**
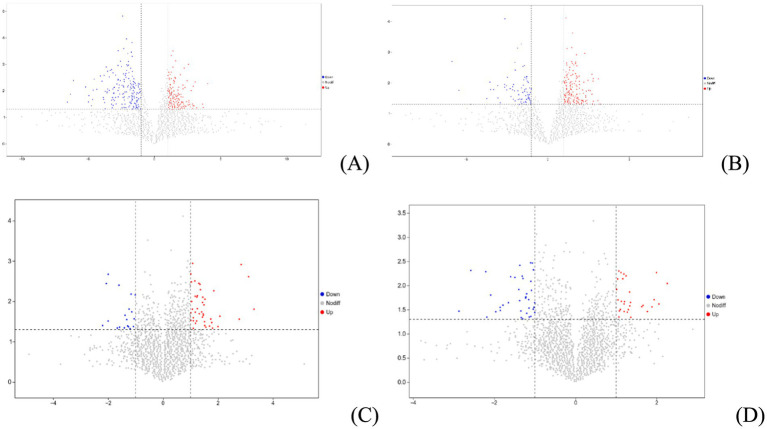
Volcano plot of metabolites from intestinal contents (**A**: identified by POS ion modes; **B**: identified by NEG ion modes) and serum samples (**C**: identified by POS ion modes; **D**: identified by NEG ion modes).

In POS ion modes, untargeted metabolomics analysis identified 252 upregulated metabolites and 227 downregulated metabolites from intestinal contents (Figure 6A) and 55 upregulated metabolites and 22 downregulated metabolites from serum (Figure 6C). In NEG ion modes, the analysis revealed 252 upregulated metabolites and 85 downregulated metabolites from intestinal contents (Figure 6B) and 31 upregulated metabolites and 38 downregulated metabolites from serum (Figure 6D).

**Figure 7:**
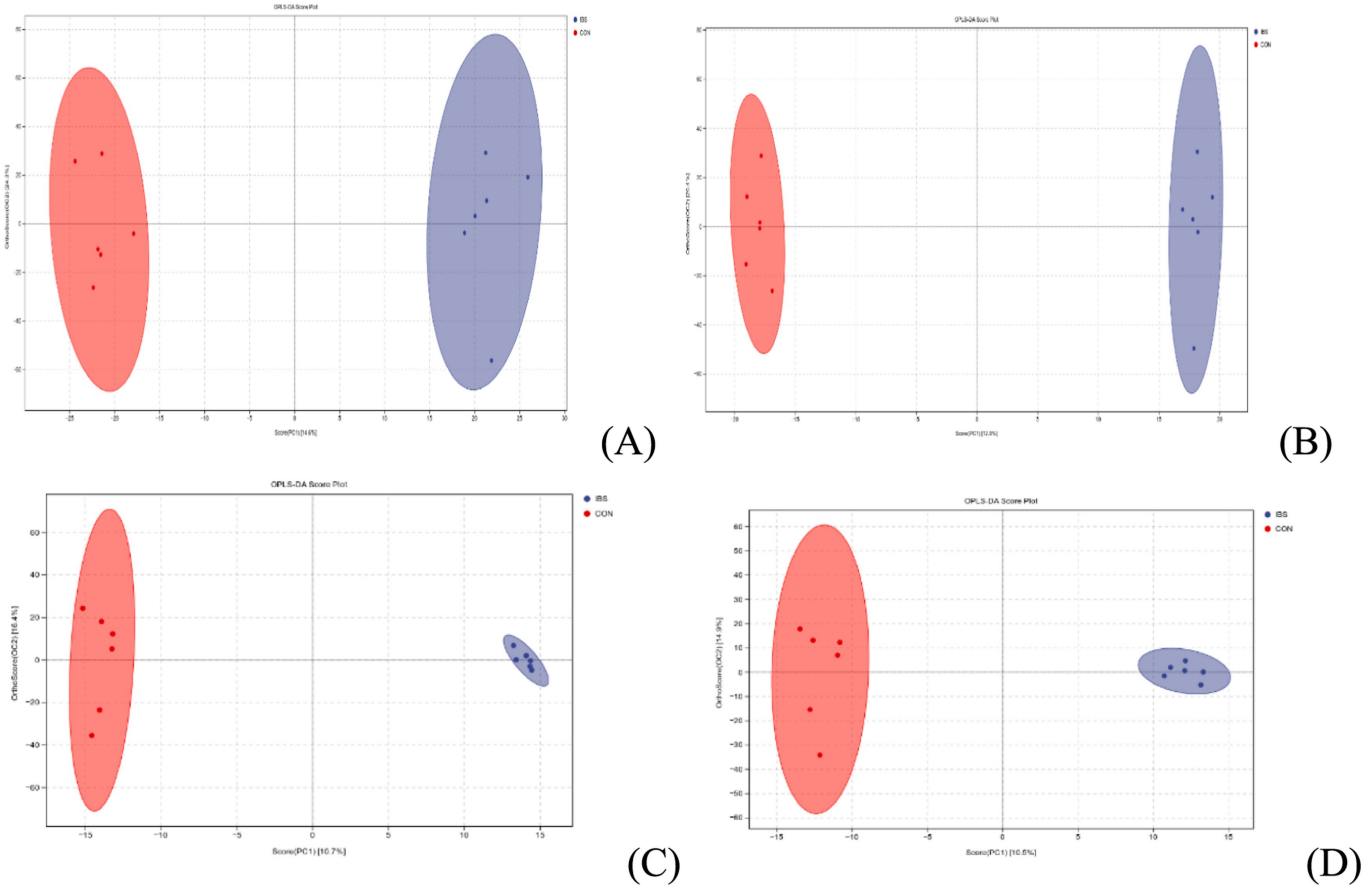
OPLS-DA analysis of metabolites from intestinal contents (**A**: identified by POS ion modes; **B**: identified by NEG ion modes) and serum samples (**C**: identified by POS ion modes; **D**: identified by NEG ion modes). CON group was not administrated with any exogenous factors IBS group was administrated with 0.30% heat-treated *B. subtilis*-derived postbiotic.

A comparison between groups was conducted using OPLS-DA analysis for intestinal contents (Figure 7A: identified in POS ion modes; Figure 7B: identified in NEG ion modes) and serum (Figure 7C: identified in POS ion modes; Figure 7D: identified in NEG ion modes). The results revealed complete separation of samples within each group, indicating the reliability of the obtained VIP values.

**Figure 8:**
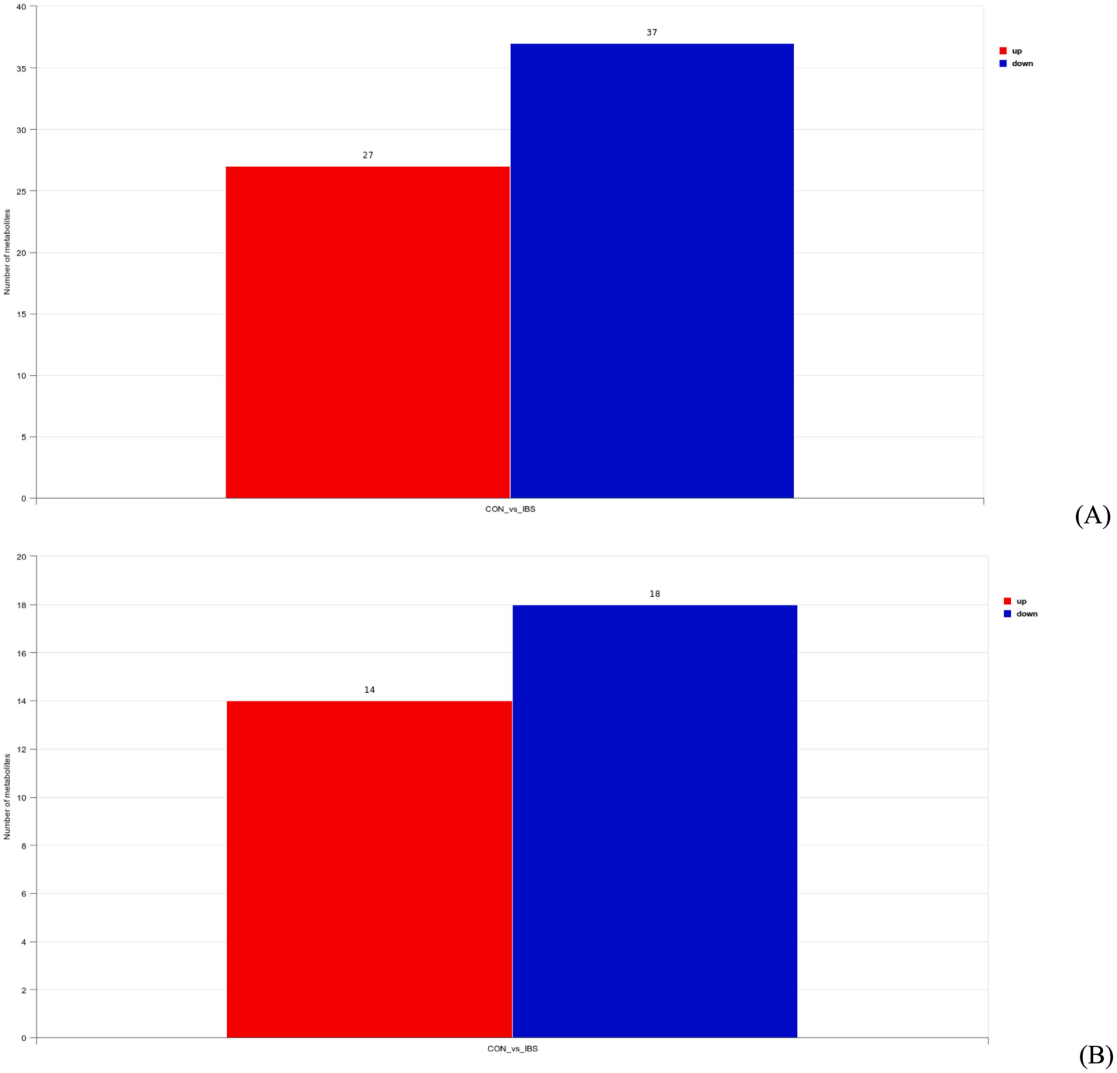
Differential metabolites from intestinal contents **(A)** and serum **(B)** samples based on a variable importance in the projection threshold of > 1 and a significance level of *p* < 0.05, in both POS and NEG ion modes. CON group was not administrated with any exogenous factors IBS group was administrated with 0.30% heat-treated *B. subtilis*-derived postbiotic.

Based on the VIP values obtained from OPLS-DA analysis, we further identified differential metabolites from intestinal contents and serum with a VIP value higher than 1 and a *p*-value lower than 0.05. The results revealed 27 upregulated and 37 downregulated differential metabolites from intestinal contents (Figure 8A) and 14 upregulated and 18 downregulated differential metabolites from serum (Figure 8B), respectively.

**Figure 9:**
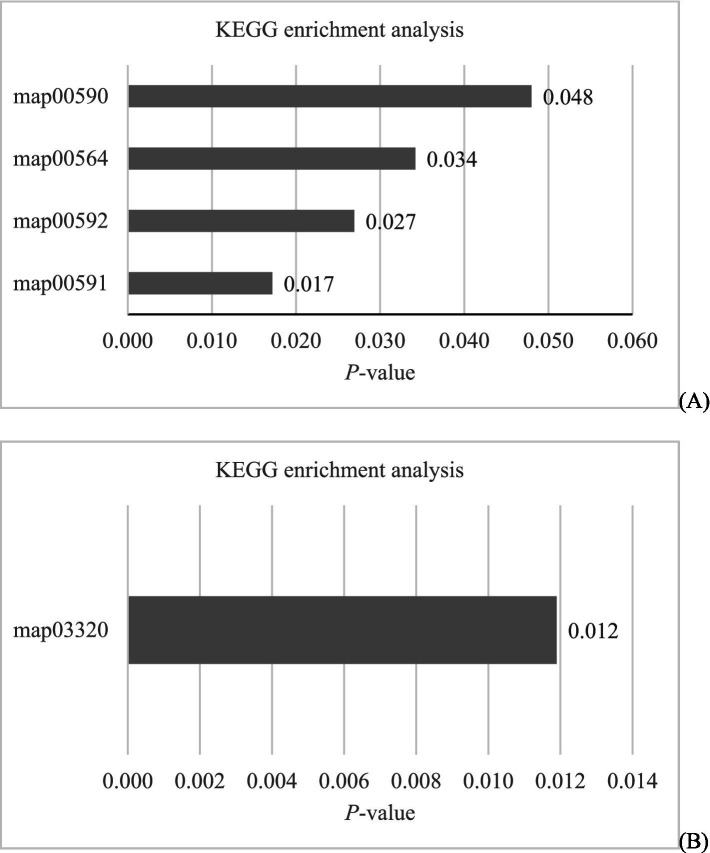
KEGG enrichment analysis of differential metabolites from intestinal contents **(A)** and serum **(B)** samples.

Further KEGG enrichment analysis for the obtained differential metabolites from intestinal contents indicated that 4 downregulated differential metabolites (PC(16:0/18:1(9Z)), PC(18:1(9Z)/18:1(9Z)), PC(18:0/18:1(9Z)), PC(14:0/16:0)) were simultaneously enriched in 4 lipid metabolism-related pathways: map00590 (*p* = 0.048), map00564 (*p* = 0.034), map00592 (*p* = 0.027), and map00591 (*p* = 0.017) (Figure 9A). For serum metabolites, the analysis indicated that 1 downregulated differential metabolite (13(S)-HODE) was enriched in the endocrine system-related pathway (map03320) with a *p*-value of 0.012 (Figure 9B).

**Figure 10:**
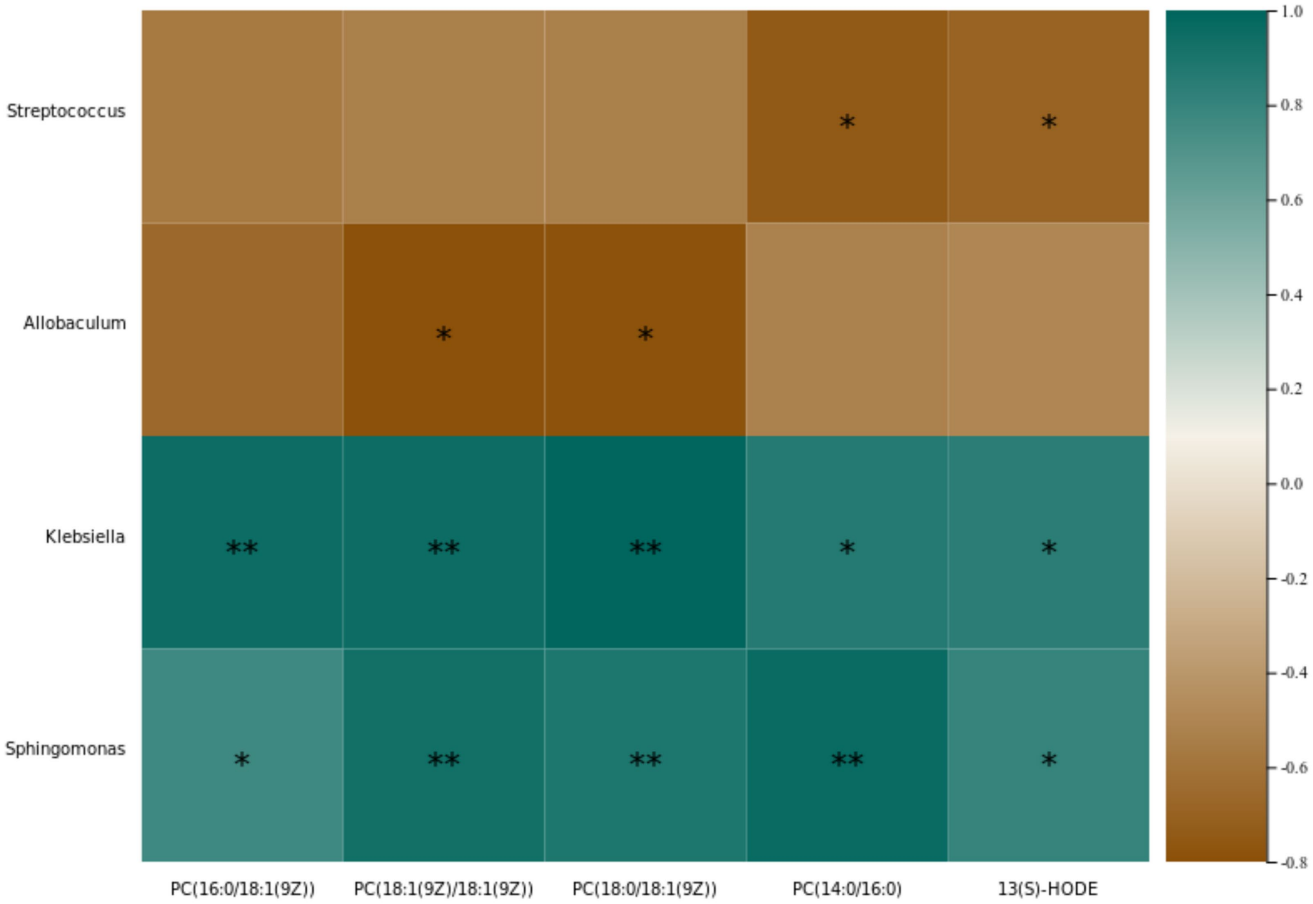
Heatmap for the Spearman correlation coefficients among identified differential bacteria and intestinal contents (PC(16:0/18:1(9Z)); PC(18:1(9Z)/18:1(9Z)); PC(18:0/18:1(9Z)); PC(14:0/16:0)) and serum (13(S)-HODE) metabolites.

We conducted a Spearman correlation analysis to explore the relationships among 4 downregulated differential metabolites from intestinal contents, 1 downregulated differential metabolite from serum, and seven differential intestinal bacteria (Figure 10). The results revealed specific correlations: content of PC(16:0/18:1(9Z)) metabolite from intestinal contents was positively correlated with *Klebsiella* (*p* = 0.004) and *Sphingomonas* (*p* = 0.033) abundance; content of PC(18:1(9Z)/18:1(9Z)) metabolite from intestinal contents showed positive correlations with *Klebsiella* (*p* = 0.004) and *Sphingomonas* (*p* = 0.005) abundance and a negative correlation with *Allobaculum* (*p* = 0.015) abundance; content of PC(18:0/18:1(9Z)) metabolite from intestinal contents exhibited positive correlations with *Klebsiella* (*p* = 0.002) and *Sphingomonas* (*p* = 0.009) abundance and a negative correlation with *Allobaculum* (*p* = 0.017) abundance; content of PC(14:0/16:0) metabolite from intestinal contents displayed positive correlations with *Klebsiella* (*p* = 0.012) and *Sphingomonas* (*p* = 0.003) abundance and a negative correlation with *Streptococcus* (*p* = 0.026) abundance; content of 13(S)-HODE metabolite from serum was positively correlated with *Klebsiella* (*p* = 0.016) and *Sphingomonas* (*p* = 0.023) abundance and negatively correlated with *Streptococcus* (*p* = 0.042) abundance (Figure 10).

**Figure 11:**
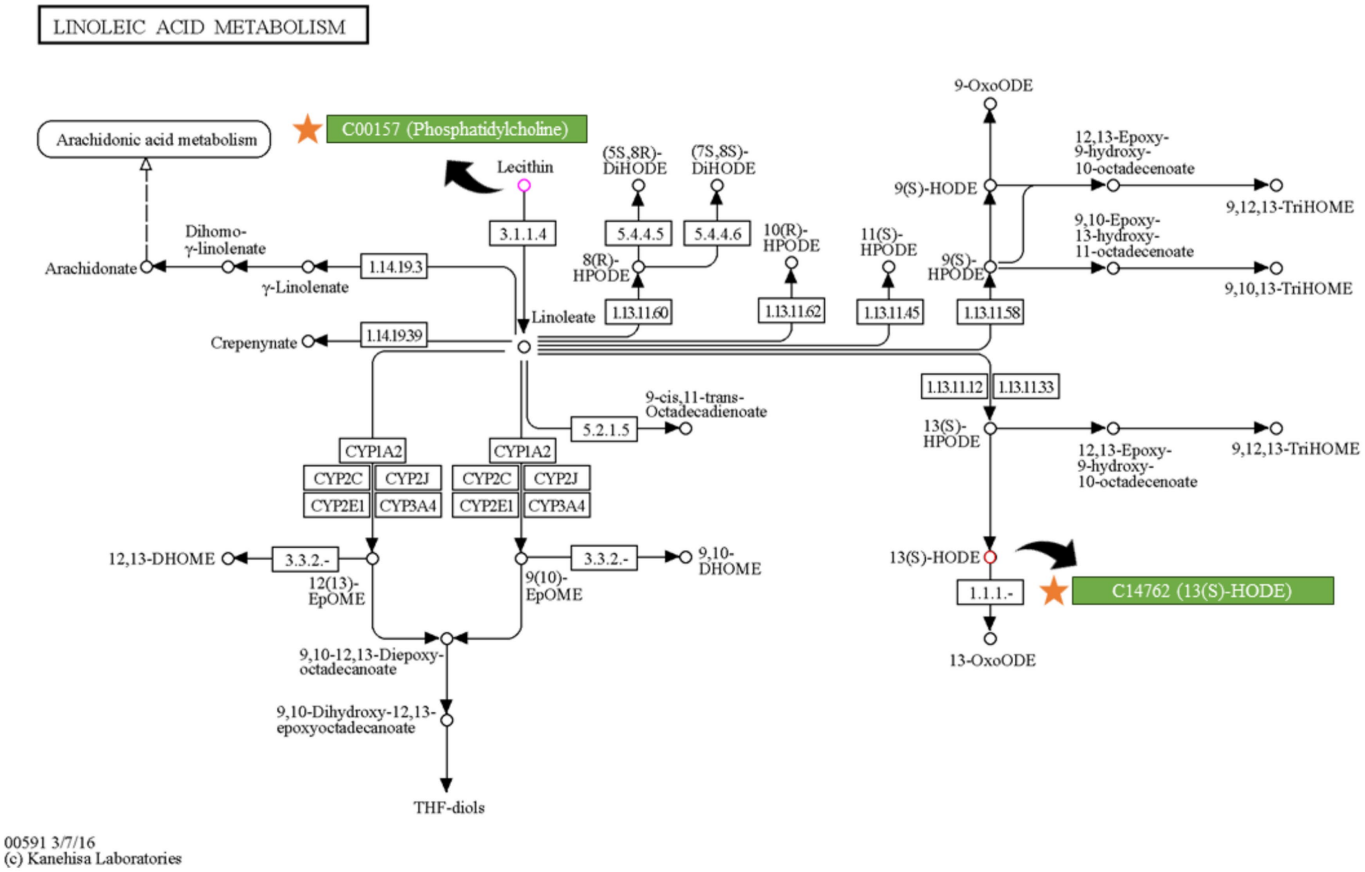
Position of key intestinal contents metabolite (C00157) and serum metabolite (C14762) in the linoleic acid metabolism pathways, diagram obtained from KEGG database (https://www.kegg.jp/pathway/map00591).

Searching for intestinal contents and serum metabolites in the KEGG database, we identified that all of the intestinal contents metabolites, PC(16:0/18:1(9Z)), PC(18:1(9Z)/18:1(9Z)), PC(18:0/18:1(9Z)), and PC(14:0/16:0), belong to the Phosphatidylcholine (C00157) compound, while the serum metabolite 13(S)-HODE belongs to the C14762 compound. These compounds were all enriched in KEGG pathway map00591, indicating shared involvement in glycerophospholipid metabolism (Figure 11).

## 4 Discussion

Sedentary behavior has been associated with increased abdominal fat accumulation, which will lead to an increased cardiovascular disease risk (25, 26). Therefore, exploring measures to reduce abdominal fat accumulation under sedentary conditions is beneficial for promoting human health. *B. subtilis* is a probiotic that may help prevent obesity. According to Lei et al. (10), the intake of *B. subtilis* B10 ameliorated abnormal lipid metabolism and oxidative stress in obese mice induced by feeding with a high fat diet. Additionally, Ayala et al. (9) noted that administering *B. subtilis* DG101 is an effective measure to control the development of obesity in diabetes patients, thereby ameliorating diabetes-related insulin resistance. However, high-temperature treated *B. subtilis*, as a postbiotic derived from *B. subtilis*, there is still limited understanding of its ability to regulate lipid metabolism. Studies investigating other probiotic-derived postbiotic administration, as conducted by Lim et al. (17), provided promising insights. They reported that administering *L. plantarum* K8-derived postbiotic reduced high fat diet feeding-induced white adipose tissue hypertrophy and hepatic fat accumulation. Watanabe et al. (15) noted that supplementing heat-killed *L. brevis* KB290 into a high fat diet was beneficial in decreasing the weight of epididymal and renal adipose tissue. In the present study, we also demonstrated that administering heat-treated *B. subtilis*-derived postbiotic decreased abdominal fat accumulation, indicating that heat-treated *B. subtilis*-derived postbiotic has suggests potential for controlling lipid accumulation.

Serum total cholesterol and triglycerides serve as key indicators associated with lipid metabolism (27). As anticipated, our study revealed that the administration of heat-treated *B. subtilis*-derived postbiotic led to a decrease in the levels of both total cholesterol and triglycerides in the serum. Similarly, Othman and Sakamoto (28) administered a diet containing heat-inactivated *B. longum* BR-108 to obese diabetic subjects and observed reductions in serum total cholesterol and triglyceride levels, along with a decrease in adipose tissue accumulation. Kikuchi et al. (14) also reported that the intake of heat-killed *B. longum* BR-108 was effective in lowering serum total cholesterol and triglyceride levels, as well as reducing epididymal body fat mass in obese mice induced by feeding with a high fat diet. Therefore, the changes in serum lipid metabolism-related parameters observed in our study further support the potential of heat-treated *B. subtilis*-derived postbiotic in controlling lipid accumulation.

However, despite the liver being a key organ in lipid metabolism, there was no discernible effect on the liver observed upon the administration of heat-treated *B. subtilis*-derived postbiotic. It is noteworthy that the relative weight of the liver also showed no adverse effects induced by the administration of heat-treated *B. subtilis*-derived postbiotic. This suggests that the administration of heat-treated *B. subtilis*-derived postbiotic does not compromise the health of the liver. This statement is supported by the observed serum parameters in this study. Common parameters for evaluating liver health include total bilirubin, gamma-glutamyl transferase, alanine aminotransferase, and total bile acids (29). In the present study, the administration of heat-treated *B. subtilis*-derived postbiotic had no significant effects on these parameters. Therefore, we consider that the administration of heat-treated *B. subtilis-*derived postbiotic has regulatory effects on lipid metabolism without impairing liver health.

On the other hand, the intestinal microbiota plays a crucial role in regulating lipid metabolism, and strategies aimed at modulating its composition are considered effective measures to control obesity development (30). In studies related to postbiotic administration, Hsieh et al. (31) found that the administration of heat-killed *L. reuteri* GMNL-263 reversed the decrease in probiotic bacteria and the increase in pathogenic bacteria in the intestine of rats induced by providing with a high fat diet, therefore ameliorating obesity-related insulin resistance and hepatic steatosis formation. Watanabe et al. (15) reported that oral intake of heat-killed *L. brevis* KB290 decreased epididymal and renal adipose tissue weights, as well as the areas of epididymal adipocytes induced by providing mice with a high fat diet, through regulating intestinal microbiota composition, and therefore suppressing insulin resistance. In our study, we observed that the administration of heat-treated *B. subtilis*-derived postbiotic induced changes in the alpha-diversity index of Pielou_e and Shannon. Pielou’s evenness index quantifies how evenly individuals are distributed among different species or taxa in a community (32). The Shannon diversity index measures the species richness and evenness (33). Therefore, administering heat-treated *B. subtilis*-derived postbiotic was beneficial in increasing the richness and evenness of the intestinal microbiota. Additionally, we observed changes in the abundance of intestinal *Bacteroides*, *Sphingomonas*, *Klebsiella*, *Streptococcus*, *Veillonella*, *Allobaculum*, and *Dorea*. Prominently, *Bacteroides* exhibited higher abundance in the intestines of overweight individuals compared to lean counterparts (34, 35). Another bacterium associated with obesity, *Sphingomonas*, displayed a positive correlation with overall and visceral fat mass (36, 37). Similarly, *Klebsiella* was found to be abundant in the intestines of obese individuals, and its presence positively correlated with serum triglyceride, total cholesterol, and low-density lipoprotein levels (38, 39). In contrast, *Streptococcus* exhibited a significant decrease in obese individuals, it possesses the potential to be used as a probiotic for obesity prevention (40, 41). Furthermore, obesity will also induce a reduction in *Veillonella* abundance, reversing its decrease is an effective measure to enhance overall body condition (42). Similarly, the abundance of *Allobaculum* decreased in obese individuals, increasing its levels will ameliorate obesity-related metabolic disorders and its abundance is negatively correlated with obesity and insulin resistance (43, 44). Additionally, the abundance of *Dorea* negative correlated with obesity, reducing its abundance can be beneficial in controlling obesity development (45, 46). In summary, characteristics of the intestinal microbiota in obese individuals include an increase in *Bacteroides*, *Sphingomonas*, and *Klebsiella*, while showing reduced levels of *Streptococcus*, *Veillonella*, *Allobaculum*, and *Dorea*. Strategies aimed at reducing the abundance of intestinal *Bacteroides*, *Sphingomonas*, and *Klebsiella*, while increasing the abundance of *Streptococcus*, *Veillonella*, *Allobaculum*, and *Dorea*, are beneficial in contribute to obesity prevention. Clearly, the administration of heat-treated *B. subtilis*-derived postbiotic represents a promising measure in this regard.

The intestinal microbiota generates various metabolites with significant regulatory functions (47). Probiotics have been reported to exert regulatory effects on lipid metabolism by producing short-chain fatty acids and secondary bile acids (48). Moreover, VanHook (49) reported that L-lactate produced by *L. paracasei* increased lipid storage in enterocytes, while acetate produced by *E. coli* decreased lipid storage and promoted lipid consumption. This underscores the regulatory impact of metabolites produced by the intestinal microbiota on lipid metabolism. In the present study, untargeted metabolomic analysis of intestinal contents identified key metabolites such as PC(16:0/18:1(9Z)), PC(18:1(9Z)/18:1(9Z)), PC(18:0/18:1(9Z)), and PC(14:0/16:0). These phosphatidylcholine metabolites were simultaneously enriched in 4 lipid metabolism-related pathways. Phosphatidylcholine and its hydrolysates are known to stimulate intestinal lipid absorption (50). Recent research has further demonstrated that intestinal *de novo* phosphatidylcholine synthesis is essential for dietary lipid absorption (51). However, we found that the administration of heat-treated *B. subtilis*-derived postbiotic resulted in a significant reduction in the amount of phosphatidylcholine in the intestinal contents. This reduction implies a decreased lipid absorption capacity for the intestine.

In addition, the serum metabolomics analysis uncovered an intriguing metabolite, 13(S)-HODE. KEGG enrichment results indicated its enrichment in the endocrine system. Notably, most of the 13(S)-HODE was incorporated into phosphatidylcholine (52). The hydrolysis of phosphatidylcholine will lead to the production of 13(S)-HODE (53). According to the pathway map from the KEGG database, the production of 13(S)-HODE from phosphatidylcholine involves two enzyme-catalyzed reactions (R07064; R03626) and one oxidation reaction. In the R07064 reaction, phosphatidylcholine reacts with water under the action of the EC 3.1.1.4 enzyme, yielding 1-Acyl-sn-glycero-3-phosphocholine and linoleate. The linoleate produced in this step undergoes oxidation in the R03626 reaction, reacting with oxygen to form 13(S)-HPODE under the action of the EC 1.13.11.12/1.13.11.33 enzyme. Subsequently, 13(S)-HPODE is rapidly reduced by peroxidases to generate 13(S)-HODE (53). It is noteworthy that, as a precursor metabolite of 13(S)-HODE, 13(S)-HPODE is not able to be absorbed by intact intestinal cells (54). However, 13(S)-HODE is rapidly taken up by endothelial cells (52). A study conducted by Zhang et al. (55) observed that 13(S)-HODE is transported into the bloodstream following gavage. This finding further supports the 13(S)-HODE uptake by intestinal cells. Thus, the transformation of phosphatidylcholine by intestinal microbiota into 13(S)-HPODE appears to occur in the inside of intestine. For metabolites derived from 13(S)-HPODE to enter the bloodstream through intestine, they must undergo an additional oxidation step to generate 13(S)-HODE. Therefore, the levels of serum 13(S)-HODE can be considered a reflection of the phosphatidylcholine content in the inside of intestine.

The Spearman correlation analysis unveiled associations between intestinal content metabolites, serum metabolites, and intestinal microbiota. Notably, *Klebsiella* and *Sphingomonas* emerged as key bacteria, with their abundance showing a positive correlation with four downregulated intestinal content metabolites and one downregulated serum metabolite. This suggests a potential link between the presence of *Klebsiella* and *Sphingomonas* and the synthesis of phosphatidylcholine. However, it is crucial to note that further evidence is required to substantiate this hypothesis.

In conclusion, the evidence presented in this manuscript supports the beneficial effects of heat-treated *B. subtilis*-derived postbiotic on reducing abdominal fat accumulation and serum total cholesterol and triglycerides levels in chickens. Furthermore, heat-treated *B. subtilis-*derived postbiotic administration decreased *Bacteroides*, *Sphingomonas*, and *Klebsiella* abundance, and increased *Streptococcus*, *Veillonella*, *Allobaculum*, and *Dorea* abundance in the intestine, aligning with strategies aimed at controlling obesity development by modulating the microbiota composition. Metabolomic analyses provide further mechanistic insights, revealing alterations in key metabolites associated with lipid metabolism pathways and endocrine system. The reduction in phosphatidylcholine (essential for intestine to absorb lipids from diet) levels in the intestine, along with changes in serum 13(S)-HODE, points to a potential impact of heat-treated *B. subtilis*-derived postbiotic administration on lipid absorption and metabolism. The Spearman correlation analysis suggests a potential link between *Klebsiella* and *Sphingomonas* bacteria and these metabolites, although further research is needed to validate their associations. The intestinal microbial structure of broiler chickens is similar to that of humans (56), so heat-treated *B. subtilis*-derived postbiotic may have the same effect on human intestinal microbes. However, there are inevitable species differences between broilers and humans, so this result still needs to be further verified. In summary, heat-treated *B. subtilis*-derived postbiotic administration promotes a more favorable microbial community, decreases an essential substance necessary for the intestine to absorb lipids from the diet, phosphatidylcholine, thereby reducing abdominal fat deposition.

## 5 Conclusion

In conclusion, the evidence presented in this manuscript supports the beneficial effects of inactivated *B. subtilis* on reducing abdominal fat deposition and serum total cholesterol and triglycerides levels in chickens. In addition, inactivated *B. subtilis* administration promotes a more favorable microbial community, decreases an essential substance necessary for the intestine to absorb lipids from the diet, phosphatidylcholine, and reducing abdominal fat accumulation in broilers.

## Data Availability

The data presented in the study are deposited in the figshare repository, accession number https://figshare.com/s/30431b58b86224fbbbfd.

## Footnotes

^1^ https://www.mzcloud.org/
