# Supplementary material for: Sedentary-related abdominal fat accumulation reduced by administrating heat-treated Bacillus subtilis-derived postbiotic
Source: Front Nutr. 2025 Jul 15;12:1612804. doi: 10.3389/fnut.2025.1612804 (PMC12303910; doi:10.3389/fnut.2025.1612804)
Supplement: Supplementary file 1 [file Table_1.DOCX]

| **Table 1.** Composition and nutrient levels of the experimental basal diet, (%, as-fed basis) | | |
| --- | --- | --- |
| Ingredients, % | Days 1-12 | Days 22-42 |
| Corn | 58.92 | 58.40 |
| Soybean meal | 31.00 | 27.10 |
| Cottonseed meal | 1.00 | 1.00 |
| Corn flour | 4.00 | 3.70 |
| Limestone | 1.00 | 0.90 |
| Calcium bicarbonate | 1.00 | 0.90 |
| NaCl | 0.25 | 0.25 |
| Sodium humate | 0.10 | 0.10 |
| Mineral and vitamin mixture^1^ | 0.30 | 0.30 |
| Lysine | 0.75 | 0.74 |
| Methionine | 0.30 | 0.25 |
| Threonine | 0.14 | 0.12 |
| Choline chloride | 0.12 | 0.12 |
| Betaine | 0.02 | 0.02 |
| Soy oil | 1.00 | 6.00 |
| Baking soda | 0.10 | 0.10 |
| Total | 100.00 | 100.00 |
| Analyzed composition, % |  |  |
| Metabolizable energy, MJ/kg | 12.13 | 12.76 |
| Crude protein | 21.25 | 20.00 |
| Crude fat | 3.50 | 7.50 |
| Calcium | 0.70 | 0.60 |
| Available phosphorus | 0.31 | 0.31 |
| Total phosphorus | 0.53 | 0.50 |
| Lysine | 1.41 | 1.35 |
| Methionine | 0.60 | 0.65 |
| Methionine + Cysteine | 0.94 | 0.99 |
| ^1^Provided per kilogram of diet: 1,500 IU retinyl acetate, 3,200 IU cholecalciferol; 10 IU _DL_-tocopheryl acetate; 0.5mg menadione sodium bisulfite; 1.8mg thiamin mononitrate; 3.6mg riboflavin; 3.5mg pyridoxine hydrochloride; 0.01mg cyanocobalamin; 0.15mg biotin; 0.55mg folic acid; 30mg nicotinic acid; 10mg pantothenic acid; 8mg copper; 0.35mg iodine; 80mg iron; 60mg manganese; 0.15mg selenium; 40mg zinc. | | |
